# Triglyceride-glucose body mass index and risk of incident venous thromboembolism: a prospective cohort study from the UK Biobank

**DOI:** 10.1186/s40001-025-03824-5

**Published:** 2026-01-10

**Authors:** Shen-Shen Huang, Shuang-Ping Li, Chai-Yi Xie, Jia-Yong Qiu, Pei-Wen Wang, Jing Zhang, Chun-Yan Cheng, Chao-Wei Ding, Yue-Jiao Ma, Dong Ding, Wan-Qing Dong, Guo-Jie Ye, Jie-Xin Zhang, Yong-Mei Zhang, Yi-Min Mao

**Affiliations:** 1 Department of Respiratory and Critical Care Medicine, The First Affiliated Hospital, and College of Clinical Medicine of Henan University of Science and Technology, Luoyang, 471003 China; 2 College of Medical Technology, Henan Medical University, Xinxiang, 453003 China; 3 Department of Cardiovascular Medicine, Guangdong Cardiovascular Institute, Guangdong Provincial People’s Hospital, Guangdong Academy of Medical Sciences, Southern Medical University, Guangzhou, 510080 China; 4 Department of Cardiology, Peking Union Medical College Hospital, Chinese Academy of Medical Sciences and Peking Union Medical College, Beijing, 100730 China; 5 Department of Respiratory and Critical Care Medicine, Xiamen Humanity Hospital Fujian Medical University, Xiamen, 361000 Fujian China; 6 Department of Endocrinology, The First Affiliated Hospital, and College of Clinical Medicine of Henan University of Science and Technology, Luoyang, 471003 China; 7 Cardiac Department, Aerospace Center Hospital, Peking University Aerospace School of Clinical Medicine, Beijing, 100049 China

**Keywords:** Venous thromboembolism, Pulmonary embolism, Deep vein thrombosis, Triglyceride-glucose body mass index, UK Biobank

## Abstract

**Background:**

Triglyceride-glucose body mass index (TyG-BMI) is an emerging surrogate indicator of insulin resistance adiposity, which has been demonstrated as a risk factor for various cardiovascular diseases including hypertension, and myocardial infarction. However, association of TyG-BMI with incident VTE remains to be investigated.

**Methods:**

This study included 328,208 participants from the prospective UK Biobank cohort without baseline VTE. The primary outcome was incident VTE, and the second outcomes were incident pulmonary embolism (PE) and deep vein thrombosis (DVT). Multivariable-adjusted Cox proportional hazards regression and restricted cubic spline (RCS) analyses assessed the association between baseline TyG-BMI and incident VTE. Stratified analyses evaluated potential effect modification by age, sex, smoking, alcohol use, diabetes, hypertension, cancer, BMI, physical activity, and dietary quality. In addition, we estimated population attributable fractions (PAFs) for elevated TyG-BMI (quartiles above quartile 1).

**Results:**

Over a median follow-up of 13.64 years, 8353 VTE events occurred. VTE incidence rose from 114.0 to 279.1 per 100,000 person-years across TyG-BMI quartiles. After full multivariable adjustment, the highest had elevated risks of VTE (HR 2.10, 95% CI 1.94–2.26), PE (HR 2.28, 95% CI 2.06–2.52), and DVT (HR 1.88, 95% CI 1.66–2.12) compared with the lowest quartile. Risk rose sharply and non-linearly beyond a TyG-BMI threshold of 231.9. Associations were stronger among women, younger participants (< 60 years), non-smokers, and individuals not on lipid-lowering therapy, and remained robust in sensitivity analyses. The PAF for VTE was 0.333 (95% CI 0.295–0.371), for PE 0.376 (0.328–0.419), and for DVT 0.276 (0.212–0.341).

**Conclusions:**

Higher TyG-BMI independently predicts increased risk of first-ever VTE, underscoring its utility as a composite biomarker for identifying individuals at heightened thrombotic risk related to metabolic dysfunction and obesity.

**Graphical abstract:**

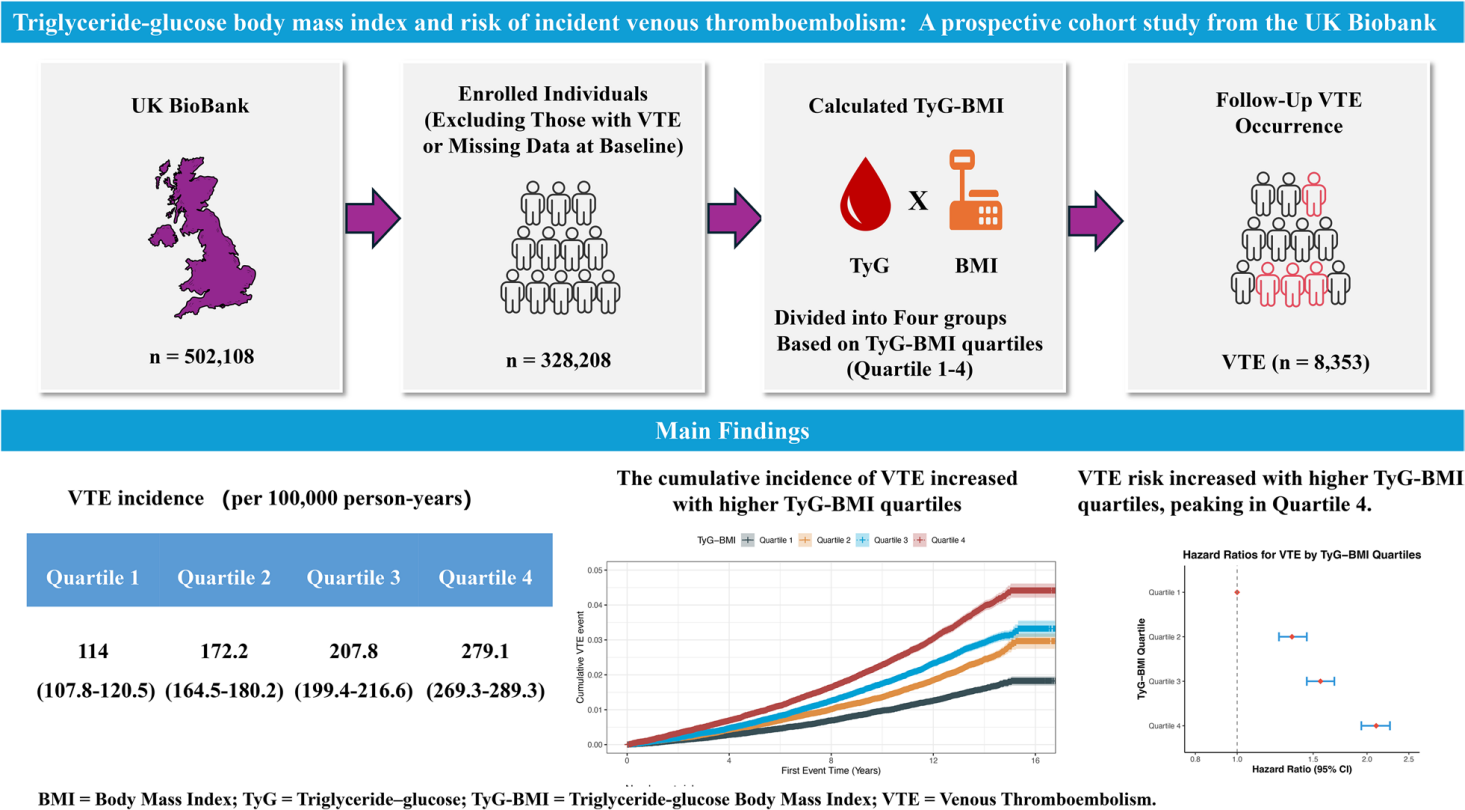

**Supplementary Information:**

The online version contains supplementary material available at 10.1186/s40001-025-03824-5.

## Introduction

Venous thromboembolism (VTE), including deep vein thrombosis (DVT) and pulmonary embolism (PE), is a major global cause of morbidity and mortality, particularly among hospitalized and elderly populations. In Western countries, its annual incidence is estimated at 1–2 cases per 1000 person-years [1, 2].In 2021, the United States reported approximately 1.22 million VTE events among hospitalized patients [3], while a nationwide study in China recorded 200,112 PE cases, with an incidence of 14.19 per 100,000 population (95% CI 14.13–14.26) [4]. VTE is now the third leading cause of vascular death, following myocardial infarction and ischemic stroke, underscoring its critical impact on cardiovascular health [1, 5, 6].

VTE is driven by both acute triggers—such as prolonged immobilization, major trauma, surgery, and active malignancy—and chronic risk factors like advanced age and inherited thrombophilias [7]. Obesity is a well-established contributor, doubling the risk compared to normal-weight individuals [8]. Prospective studies further demonstrate that elevated body mass index (BMI) and central (abdominal) adiposity exhibit stronger associations with VTE incidence than with coronary heart disease, underscoring the unique prothrombotic effect of visceral fat distribution [9]. These observations have spurred growing interest in metabolic contributions to VTE. Metabolic syndrome—defined by abdominal obesity, hypertension, hyperglycemia, hypertriglyceridemia, and low HDL cholesterol—creates a systemic proinflammatory and prothrombotic milieu [10]. In a landmark case–control study, 35% of patients with unprovoked recurrent VTE met criteria for metabolic syndrome, versus 20% of controls, suggesting a twofold increased risk [10]. Similarly, the Tromso Study reported a 65% higher VTE risk in those with metabolic syndrome, though only obesity remained an independent predictor after multivariable adjustment [11].

While metabolic syndrome and central obesity are linked to VTE, BMI alone may not fully capture metabolic risk. Around 17% of obese individuals are insulin sensitive—a phenotype known as metabolically healthy obesity—highlighting the limits of using weight-based measures alone [12]. Importantly, insulin resistance can promote thrombosis even without obesity, underscoring the need for better markers that reflect both metabolic and thrombotic risk [13].

The triglyceride-glucose (TyG) index, derived from fasting triglyceride and glucose levels, is a simple surrogate for insulin resistance [14]. When combined with BMI, the resulting TyG-BMI index more accurately reflects underlying metabolic dysfunction [15]. Previous studies have validated TyG-BMI as a reliable marker for insulin resistance in non-diabetic cohorts [16], showing strong associations with type 2 diabetes, hypertension, and cardiovascular disease [17–19]. By integrating markers of dysglycemia, dyslipidemia, and obesity, TyG-BMI serves as a comprehensive indicator of metabolic risk in population-based research.

Given its ability to capture both insulin resistance and adiposity, TyG-BMI may reflect a prothrombotic metabolic state. While its associations with cardiometabolic diseases are well established, [18, 19] whether TyG-BMI is linked to VTE remains unclear. Therefore, this study aims to evaluate the prospective relationship between baseline TyG-BMI and incident VTE, including DVT and PE, using data from the UK Biobank, which could enhance risk stratification algorithms and inform targeted prevention and management strategies for VTE in high-risk populations.

## Methods

### Study design and participants

The UK Biobank is a large, population-based, prospective cohort that recruited 502,218 volunteers aged 37–73 years throughout the United Kingdom between 2006 and 2010 by means of touchscreen questionnaires and nurse-led interviews. These baseline assessments captured extensive demographic, socioeconomic, lifestyle, and health-related information. Follow-up for health outcomes is ongoing; the data freeze used for the present analysis included events recorded up to 31 March 2023. UK Biobank received ethical approval from the North West Multi-centre Research Ethics Committee (reference 11/NW/03820), and all participants provided written informed consent. Access to the resource is available to bona fide researchers via the online Access Management System (AMS). The present study was performed under application number 663576.

### Case definition of venous thromboembolism

**Fig. 1:**
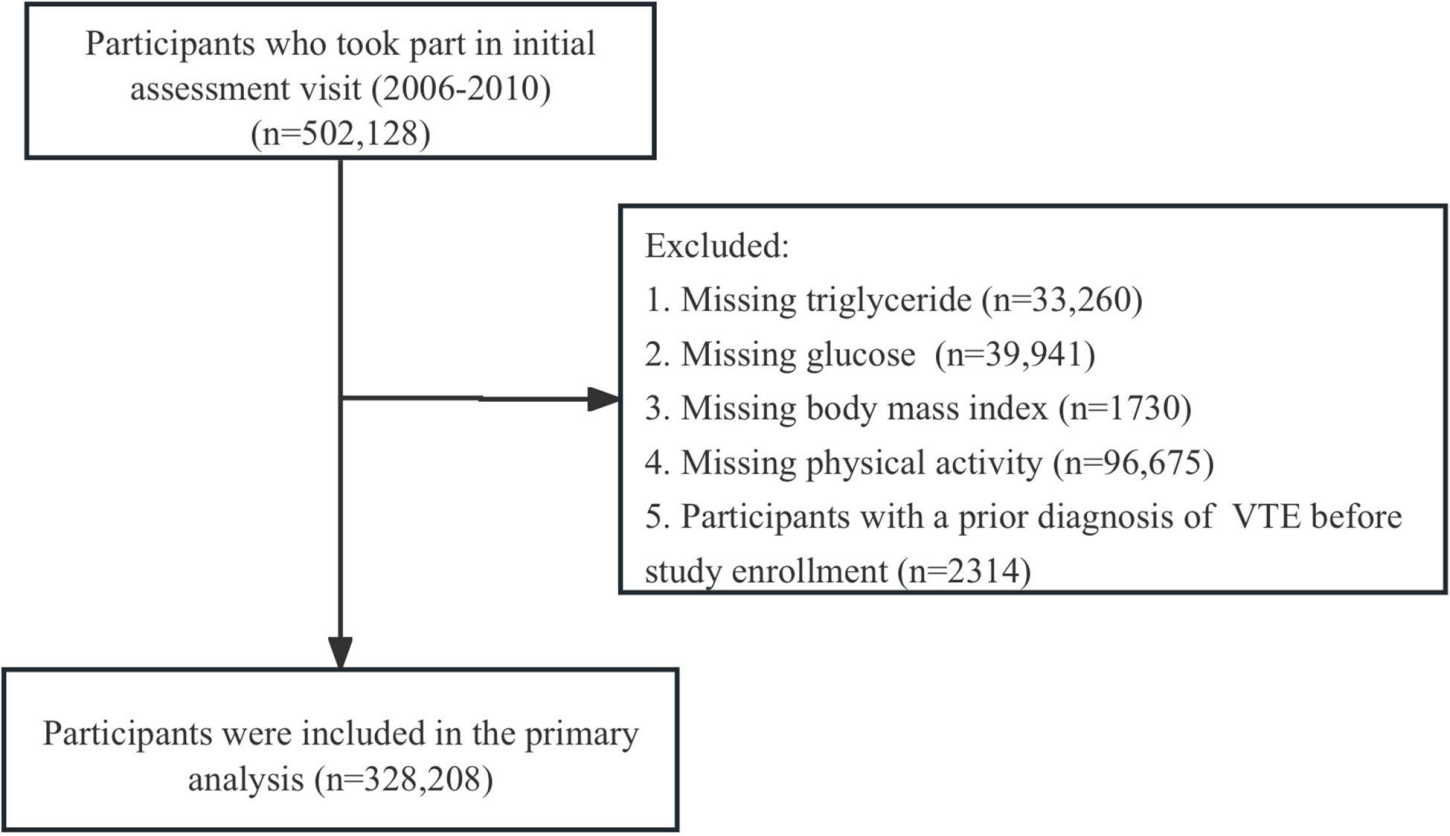
Flowchart of participant selection. VTE, Venous thromboembolism

First-ever hospital admissions coded for VTE were identified through linkage to national inpatient records. VTE diagnoses were defined with the 10th revision of the International Classification of Diseases (ICD-10) using the following codes: I82.4, I82.5, I80.1, I80.2, I80.3, I80.9, I82.8, and I82.9 for DVT, and I26.0 and I26.9 for PE [20]. Participants with any record of VTE before baseline assessment were excluded. Additional exclusions were individuals missing baseline measurements of triglycerides (TG), fasting blood glucose (FBG), BMI, or physical activity. After applying the exclusion criteria, 328,208 eligible participants remained in the analytic cohort. The TyG-BMI index was calculated for each individual at baseline and the cohort was categorized into quartiles of this index for subsequent analyses (Fig. 1).

### Measures

From the core UK Biobank database, we extracted sociodemographic data (age, sex, ethnicity, and Townsend Deprivation Index [TDI]), lifestyle factors (smoking status, alcohol consumption, diet, physical activity, and BMI), physician-diagnosed comorbidities (diabetes and hypertension), and information on medication use (lipid-lowering agents and insulin). The TDI is a composite area-based measure of material deprivation derived from UK census output areas prior to participant recruitment, combining four census variables: unemployment, household overcrowding, non-car ownership, and non-owner-occupied households, linked to each participant by postcode (UK Biobank Data-Field 189). Ethnicity was defined using self-reported “Ethnic background” at baseline (UK Biobank Data-Field 21,000), with major categories such as White, Black or Black British, Asian or Asian British, and Other or Mixed as per UK Biobank coding. Anthropometry (height and weight), resting systolic blood pressure (SBP) and diastolic blood pressure (DBP), and laboratory assays, such as Hemoglobin A1c (HbA1c), fasting blood glucose (FBG), triglycerides (TG), total cholesterol (TC), high-density lipoprotein cholesterol (HDL-C), and low-density lipoprotein cholesterol (LDL-C), were obtained at baseline assessment. Smoking status was classified as never, previous or current in accordance with UK Biobank field 20116, whereas alcohol intake status was also coded as never, previous or current. “Healthy activity” was defined as meeting the World Health Organization’s aerobic guidelines—namely, at least 2.5 h per week of moderate-intensity activity, at least 1.25 h per week of vigorous-intensity activity, or an equivalent combination of the two [21]. “Healthy diet” was defined by adherence to at least five of nine cardiovascular-health dietary targets: increased consumption of fruits, vegetables, whole grains, fish or shellfish, dairy products, and vegetable oils, and reduced (or no) consumption of refined grains, processed or unprocessed meats, and sugar-sweetened beverages [22].

### Outcome definition

The primary endpoint was incident VTE; secondary endpoints were PE and DVT, analyzed separately. The date of first hospital admission bearing a qualifying ICD-10 code was taken as the event date (I26.0, I26.9 for PE; I80.1, I80.2, I80.3, I80.9, I82.4, I82.5, I82.8, I82.9 for DVT) [20]. Person-time accrued from baseline until the earliest of VTE diagnosis, death, loss to follow-up, or 31 May 2024.

### Exposure calculation: TyG-BMI index

The triglyceride-glucose (TyG) index was computed as ln [FBG (mg・dl⁻^1^) × TG (mg・dl⁻^1^)/2] [14]. Body mass index (BMI) was calculated as weight (kg) divided by height squared (m^2^). The composite TyG-BMI index was defined as TyG × BMI, following prior literature [18].

### Statistical analyses

Missing values were < 10% for all covariates. Categorical variables with missingness were imputed using mode substitution, whereas continuous variables (HbA1c, BMI, SBP, DBP, TC, HDL-C, LDL-C) were replaced with mean values. Participants were stratified into quartiles of baseline TyG-BMI. Normality of continuous variables was assessed with the Anderson–Darling test. Age, SBP, DBP, FBG, and TG are reported as mean (interquartile range) and compared across quartiles using the Kruskal–Wallis test. Approximately normally distributed variables (such as HbA1c, BMI, TC, HDL-C, LDL-C, RC, and TyG-BMI) are summarized as mean ± standard deviation and compared with one-way ANOVA. Categorical variables are presented as number (percentage) and evaluated with Pearson’s *χ*^2^ test.

Incidence rates of VTE, PE, and DVT were estimated using Poisson regression and are reported per 100 000 person-years with 95% confidence intervals (CIs). Cumulative incidence functions were visualized with Kaplan–Meier curves and compared by log-rank testing [20]. Hazard ratios (HRs) and 95% CIs across TyG-BMI quartiles were estimated using Cox proportional-hazards regression. Model 1 adjusted for age and sex; model 2 further adjusted for smoking and alcohol status; model 3 additionally controlled for ethnicity, Townsend deprivation index, diabetes, apolipoprotein A (ApoA), systolic blood pressure (SBP), HbA1c, healthy diet, hypertension, LDL-C, C-reactive protein (CRP), use of lipid-lowering drugs, insulin use, healthy physical activity, and RC. In addition, TyG-BMI was z-standardized and modeled as a continuous predictor; per-1 SD hazard ratios were estimated from a Cox model adjusted for the same covariates as Model 3. Potential non-linearity was explored with restricted cubic splines fitted in model 3 [23].

To mitigate reverse causation and protopathic bias inherent to single-baseline cohort designs, we conducted three prespecified landmark sensitivity analyses that excluded incident VTE occurring within the first 1, 3, and 5 years of follow-up. Excluding early follow-up is a standard approach in cardiovascular epidemiology to reduce bias from occult disease processes and short-term precipitants clustered near baseline [24]. We modeled the relationship between continuous TyG-BMI and incident VTE using restricted cubic splines (RCS) in Cox proportional hazards models. We used 3 knots, placed at the 10th, 50th, and 90th percentiles of the TyG-BMI distribution, following Harrell’s recommended default placements. We tested for non-linearity via likelihood ratio test comparing spline vs. linear term. CIs around the spline curve were plotted. We calculated sensitivity, specificity, positive predictive value (PPV), and negative predictive value (NPV) of administrative-code–based VTE ascertainment stratified by TyG-BMI quartiles, using Quartile 1 as reference. These metrics were computed based on 2 × 2 classification (event vs. non-event) across follow-up, with true status defined by administrative records and “test positive” defined by being in a higher TyG-BMI quartile. Effect modification was prespecified and assessed both by testing multiplicative interaction terms and by conducting stratified analyses across the following subgroups: age (< 60 vs. ≥ 60 years), sex (male vs. female), ethnicity (White/Black/Asian vs. other), smoking status (never/former vs. current), alcohol consumption (non-drinkers vs. drinkers), diabetes (absent vs. present), hypertension (absent vs. present), cancer (absent vs. present), BMI (< 30 vs. ≥ 30 kg/m^2^), heathy activity (no vs. yes), and diet quality (healthy diet vs. unhealthy diet).

We estimated population attributable fractions (PAFs) for TyG-BMI overall and in key subgroups using adjusted hazard ratios (HRs) from Cox models and subgroup-specific exposure prevalence across TyG-BMI quartiles (Quartile 1–Quartile 4), with Quartile 1 as the counterfactual reference. For multi-category exposure, PAF was computed as ${\mathrm{PAF}}=\frac{{\sum }_{i=2}^{4}{p}_{i}\hspace{0.17em}\left(H{R}_{i}-1\right)}{1+{\sum }_{i=2}^{4}{p}_{i}\hspace{0.17em}\left(H{R}_{i}-1\right)}$, 95% CIs were obtained via non-parametric bootstrap (1,000 resamples), re-fitting the Cox model and re-estimating the within-sample p_i_ at each iteration [25]. Subgroup PAFs were estimated within each stratum (e.g., sex, age, ethnicity, lipid-lowering therapy use), using the same covariate set excluding the stratifying variable to avoid overadjustment. These PAFs should be interpreted as population burden measures that depend on both association strength (HR) and exposure prevalence, rather than as effects of subgroup membership or treatment efficacy. All analyses were performed using R, version 4.4.3. Two-sided *p*-values < 0.05 were considered statistically significant.

## Results

### Baseline characteristic

Table 1 summarizes baseline characteristics of the 323,208 eligible participants (mean age 57 years). Those in the highest TyG-BMI quartile were significantly older and more likely to be male (*P* < 0.001). Higher TyG-BMI was associated with adverse cardiometabolic profiles, including higher levels of HbA1c, BMI, SBP, DBP, FBG, TG, TC, and LDL-C, and lower levels of HDL-C (all *P* < 0.001). They were also more likely to be former or current smokers, never or former drinkers, to have physician-diagnosed diabetes or hypertension, and to be receiving lipid-lowering agents or insulin (all *P* < 0.001).

**Table 1:** Baseline characteristics of participants

Characteristic	Total (*n* = 328,208)	TyG-BMI				*P*-value
		Quartile 1 < 203.6 (*n* = 82,052)	Quartile 2 203.6–231.9 (*n* = 82,052)	Quartile 3 231.9–265.2 (*n* = 82,052)	Quartile 4 ≥ 265.2 (*n* = 82,052)	
Age, years	57.0 (50.0, 63.0)	55.0 (47.0, 62.0)	58.0 (50.0, 63.0)	59.0 (51.0, 64.0)	58.0 (51.0, 63.0)	< 0.001
Male, *n* (%)	158,957(48.4)	25,589 (31.2)	39,974 (48.7)	47,657 (58.1)	45,737 (55.7)	< 0.001
Ethnicity, *n* (%)						
White	312,599 (95.2)	78,329 (95.5)	78,219 (95.3)	77,961 (95)	78,107 (95.2)	< 0.001
Black	4591 (1.4)	878 (1.1)	1044 (1.3)	1281 (1.6)	1393 (1.7)	
Asian	6496 (2.0)	1644 (2.0)	1708 (2.1)	1752 (2.1)	1379 (1.7)	
Other	4522 (1.4)	1201 (1.5)	1081 (1.3)	1058 (1.3)	1173 (1.4)	
TDI	− 2.3 (− 3.7, 0.3)	− 2.3 (− 3.8, 0.1)	− 2.4 (− 3.8, − 0.1)	− 2.3 (− 3.7, 0.2)	− 1.9 (− 3.5, 0.9)	< 0.001
BMI, kg/m^2^	26.6 (24.1, 29.7)	22.5 (21.3, 23.6)	25.4 (24.5, 26.4)	27.9 (26.9, 29.1)	32.3 (30.5, 35.0)	< 0.001
SBP, mmHg	138 (125, 151)	130 (118, 144)	137 (125, 150)	140 (129, 154)	143 (131, 156)	< 0.001
DBP, mmHg	82 (75, 89)	77 (71, 84)	81 (74, 88)	83 (77, 90)	86 (79, 93)	< 0.001
Smoking status, *n* (%)						
Never	179,894 (54.8)	49,870 (60.8)	46,330 (56.5)	43,096 (52.5)	40,576 (49.5)	< 0.001
Previous	115,217 (35.1)	23,952 (29.2)	27,712 (33.8)	30,575 (37.3)	32,989 (40.2)	
Current	33,097 (10.1)	8230 (10.0)	8010 (9.8)	8381 (10.2)	8487 (10.3)	
Drinking status, *n* (%)						
Never	12,402 (3.8)	2879 (3.5)	2825 (3.4)	2959 (3.6)	3750 (4.6)	< 0.001
Previous	11,011 (3.4)	2556 (3.1)	2342 (2.9)	2653 (3.2)	3465 (4.2)	
Current	304,795 (92.9)	76,617 (93.4)	76,885 (93.7)	76,440 (93.2)	74,837 (91.2)	
Diabetes, *n* (%)	16,170 (4.9)	1082 (1.3)	1911 (2.3)	3381 (4.1)	9798 (11.9)	< 0.001
Hypertension, *n* (%)	91,440 (27.9)	11,105 (13.5)	18,489 (22.5)	25,329 (30.9)	36,512 (44.5)	< 0.001
Cancer, *n* (%)	28,744 (8.8)	7419 (9.0)	7248 (8.8)	7133 (8.7)	6944 (8.5)	< 0.001
Insulin	3318 (1.0)	431 (0.5)	502 (0.6)	634 (0.8)	1753 (2.1)	< 0.001
Lipid-lowering drugs, *n* (%)	55,115 (16.8)	6028 (7.3)	11,016 (13.4)	15,775 (19.2)	22,291 (27.2)	< 0.001
Healthy diet	124,802 (38.0)	36,414 (44.4)	32,580 (39.7)	29,243 (35.6)	26,535 (32.3)	< 0.001
Healthy activity	268,525 (81.8)	70,744 (86.2)	69,153 (84.3)	67,037 (81.7)	61,591 (75.1)	< 0.001
TG, mmol/L	1.5 (1.0, 2.1)	1.0 (0.8, 1.2)	1.3 (1.0, 1.8)	1.7 (1.3, 2.3)	2.2 (1.6, 3.1)	< 0.001
FBG, mmol/L	4.9 (4.6, 5.3)	4.8 (4.5, 5.1)	4.9 (4.6, 5.2)	5.0 (4.6, 5.3)	5.1 (4.7, 5.6)	< 0.001
HDL-C, mmol/L	1.4 (1.2, 1.7)	1.7 (1.4, 1.9)	1.5 (1.3, 1.7)	1.3 (1.1, 1.5)	1.2 (1.0, 1.4)	< 0.001
LDL-C, mmol/L	3.5 (2.9, 4.1)	3.3 (2.8, 3.8)	3.6 (3.0, 4.1)	3.7 (3.1, 4.3)	3.6 (3.0, 4.2)	< 0.001
TC, mmol/L	5.6 (4.9, 6.4)	5.5 (4.8, 6.2)	5.7 (5.0, 6.4)	5.8 (5.0, 6.5)	5.7 (4.8, 6.5)	< 0.001
ApoB, g/L	1.0 (0.9, 1.2)	0.9 (0.8, 1.1)	1.0 (0.9, 1.2)	1.1 (0.9, 1.2)	1.1 (0.9, 1.2)	< 0.001
ApoA, g/L	1.5 (1.3, 1.7)	1.6 (1.5, 1.8)	1.5 (1.4, 1.7)	1.5 (1.3, 1.6)	1.4 (1.3, 1.6)	< 0.001
CRP, mg/L	1.3 (0.6, 2.6)	0.7 (0.4, 1.3)	1.1 (0.6, 2.0)	1.5 (0.8, 2.7)	2.4 (1.3, 4.5)	< 0.001
HbA1c, mmol/mol	35.1 (32.6, 37.7)	34.1 (31.9, 36.3)	34.6 (32.3, 37.0)	35.2 (32.9, 37.8)	36.7 (34.0, 40.1)	< 0.001
RC, mmol/L	0.6 (0.5, 0.8)	0.5 (0.4, 0.6)	0.6 (0.5, 0.8)	0.7 (0.6, 0.9)	0.8 (0.6, 1.0)	< 0.001
TyG	8.7 (8.3, 9.1)	8.2 (8.0, 8.5)	8.6 (8.3, 8.8)	8.8 (8.6, 9.1)	9.1 (8.8, 9.5)	< 0.001
TyG-BMI	231.9 (203.6, 265.2)	186.7 (174.7, 195.8)	217.9 (211.0, 224.8)	247.0 (239.2, 255.4)	293.0 (277.2, 319.3)	< 0.001

*TDI* Townsend Deprivation Index, *BMI* Body Mass Index, *SBP* Systolic Blood Pressure, *DBP* Diastolic Blood Pressure, *TyG* Triglyceride-glucose index, *TyG-BMI* Triglyceride-glucose index multiplied by BMI, *RC* Remnant Cholesterol, *TG* Triglycerides, *FBG* Fasting Blood Glucose, *HDL-C* High-Density Lipoprotein Cholesterol, *LDL-C* Low-Density Lipoprotein Cholesterol, *TC* Total Cholesterol, *ApoB* Apolipoprotein B, *ApoA* Apolipoprotein A, *CRP* C-reactive protein, *HbA1c* Hemoglobin A1c, *Insulin* Use of insulin, Lipid-lowering drugs, Use of lipid-lowering medications

### Association between TyG-BMI and incident VTE

**Fig. 2:**
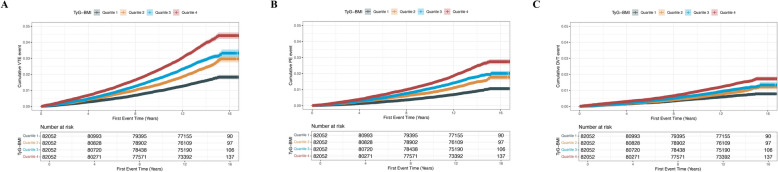
Kaplan–Meier curves of cumulative incidence. **A** VTE, **B** PE, **C** DVT. TyG-BMI index quartile 1 was used as the reference group. *TyG-BMI* triglyceride-glucose body mass index, *DVT* deep vein thrombosis, *PE* pulmonary embolism, *VTE* venous thromboembolism

**Fig. 3:**
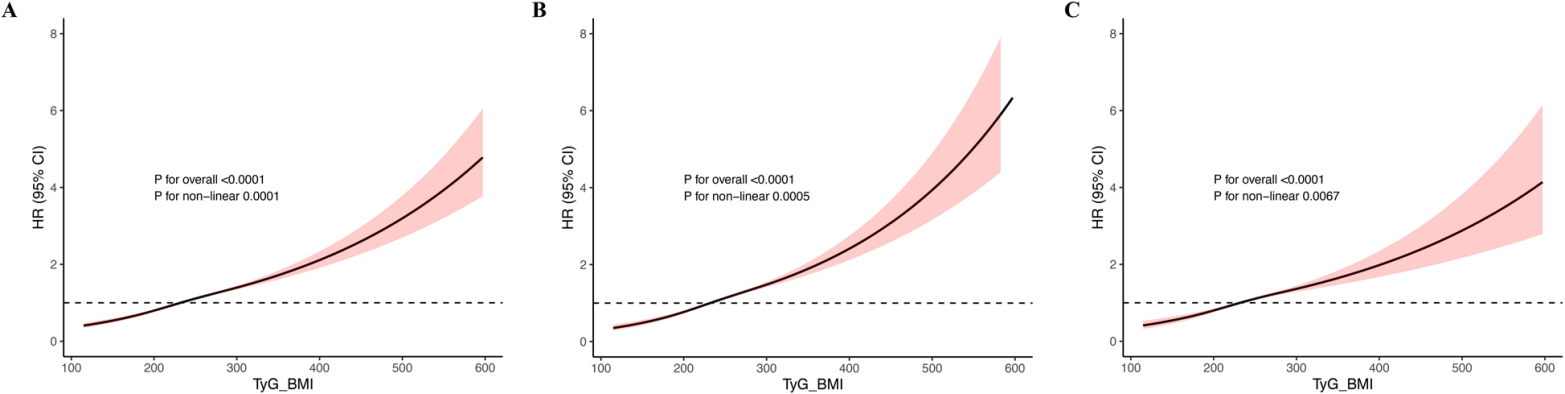
Non-linear relationship between TyG-BMI index and incident risk of outcomes. **A** TyG-BMI index in VTE, **B** TyG-BMI index in PE, **C** TyG-BMI index in DVT. The relationship was evaluated by RCS after adjustment for age, sex, smoking status, drinking status, ethnicity, Townsend deprivation index, diabetes, ApoA, SBP, HbA1c, healthy diet, hypertension, LDL-C, CRP, lipid-lowering drugs, insulin use, healthy physical activity, and RC (model 3). Solid lines in the figure represent the HRs, and the shaded regions represent the 95% CIs. *TyG-BMI* triglyceride-glucose body mass index, *DVT* deep vein thrombosis, *PE* pulmonary embolism, *VTE* venous thromboembolism

During a median follow-up of 13.64 years (interquartile range 12.87–14.31 years), 8353 first VTE events were documented, including 5026 PE and 3327 DVT cases. Cumulative incidence of VTE increased progressively with higher TyG-BMI quartiles (Fig. 2). Restricted cubic spline analysis revealed a significant non-linear association between TyG-BMI and thrombotic risk, with risk rising gradually beyond a TyG-BMI value of 231.9 (*P* for non-linearity < 0.001) (Fig. 3), suggesting a potential inflection point beyond which prothrombotic risk accelerates. VTE incidence rose progressively across TyG-BMI quartiles, from 114.0 (107.8–120.5) to 279.1 (269.3–289.3) per 100,000 person-years. Similar trends were observed for PE (63.5 to 171.9) and DVT (50.7 to 108.8). In unadjusted analyses, the highest quartile had over twice the risk of VTE (HR 2.47), PE (HR 2.73), and DVT (HR 2.15) versus the lowest. After full adjustment for age, sex, smoking, drinking status, ethnicity, Townsend deprivation index, diabetes, ApoA, SBP, HbA1c, healthy diet, hypertension, LDL-C, CRP, lipid-lowering drugs, insulin, healthy physical activity, and RC (Model 3), risks remained significantly elevated: VTE (HR 2.10), PE (HR 2.28), and DVT (HR 1.88).

A clear dose–response was observed (*P* for trend < 0.001). When analyzed continuously, each standard-deviation (SD) increase in TyG-BMI conferred HRs of 1.31 (1.28–1.34) for VTE, 1.34 (1.30–1.39) for PE, and 1.27 (1.22–1.32) for DVT. In the sensitivity analyses, we performed three separate analyses by excluding all incident VTEs occurring within the first 1 year, first 3 years, and first 5 years of follow-up, respectively; the associations remained consistent (Fig. S1-3). The sensitivity analyses excluding early events (within 1, 3, or 5 years) confirmed the robustness of significant trend by TyG-BMI quartile (*P* < 0.001) (Table 2).

**Table 2:** Baseline TyG-BMI index and incident risks of VTE

	TyG-BMI				*P* for trend	Per SD	P-value
	Quartile 1	Quartile 2	Quartile 3	Quartile 4			
Outcome: VTE							
Number of events	1247	1871	2248	2987			
Persons	82,052	82,052	82,052	82,052			
Person-years	1,094,323	1,086,685	1,081,744	1,070,103			
Incidence	114.0 (107.8–120.5)	172.2 (164.5–180.2)	207.8 (199.4–216.6)	279.1 (269.3–289.3)			
Unadjusted model	Reference	1.51 (1.41–1.63)	1.83 (1.71–1.96)	2.47 (2.31–2.63)	< 0.001	1.34 (1.31–1.36)	< 0.001
Model 1	Reference	1.31 (1.22–1.40)	1.51 (1.41–1.62)	2.11 (1.97–2.25)	< 0.001	1.32 (1.29–1.34)	< 0.001
Model 2	Reference	1.30 (1.21–1.40)	1.50 (1.40–1.61)	2.07 (1.93–2.21)	< 0.001	1.31 (1.28–1.33)	< 0.001
Model 3	Reference	1.34 (1.25–1.45)	1.56 (1.45–1.68)	2.10 (1.94–2.26)	< 0.001	1.31 (1.28–1.34)	< 0.001
Sensitivity analysis 1	Reference	1.33 (1.23–1.43)	1.55 (1.44–1.67)	2.07 (1.91–2.24)	< 0.001	1.31 (1.27–1.34)	< 0.001
Sensitivity analysis 2	Reference	1.34 (1.24–1.45)	1.57 (1.45–1.70)	2.08 (1.92–2.27)	< 0.001	1.30 (1.27–1.34)	< 0.001
Sensitivity analysis 3	Reference	1.35 (1.24–1.47)	1.59 (1.46–1.73)	2.10 (1.92–2.30)	< 0.001	1.30 (1.27–1.34)	< 0.001
Outcome: PE							
Number of events	695	1119	1373	1839			
Persons	82,052	82,052	82,052	82,052			
Person-years	1,094,323	1,086,685	1,081,744	1,070,103			
Incidence	63.5 (59.0–68.4)	103.0 (97.1–109.2)	126.9 (120.4–133.8)	171.9 (164.2–179.9)			
Unadjusted model	Reference	1.63 (1.48–1.79)	2.01 (1.83–2.20)	2.73 (2.50–2.97)	< 0.001	1.37 (1.33–1.40)	< 0.001
Model 1	Reference	1.40 (1.27–1.54)	1.66 (1.51–1.82)	2.33 (2.14–2.55)	< 0.001	1.35 (1.32–1.38)	< 0.001
Model 2	Reference	1.40 (1.27–1.54)	1.65 (1.50–1.81)	2.30 (2.10–2.51)	< 0.001	1.34 (1.31–1.38)	< 0.001
Model 3	Reference	1.42 (1.29–1.57)	1.68 (1.53–1.86)	2.28 (2.06–2.52)	< 0.001	1.34 (1.30–1.39)	< 0.001
Sensitivity analysis 1	Reference	1.41 (1.28–1.55)	1.68 (1.52–1.86)	2.27 (2.05–2.52)	< 0.001	1.34 (1.30–1.38)	< 0.001
Sensitivity analysis 2	Reference	1.38 (1.25–1.53)	1.65 (1.49–1.83)	2.24 (2.01–2.49)	< 0.001	1.33 (1.29–1.38)	< 0.001
Sensitivity analysis 3	Reference	1.37 (1.23–1.53	1.65 (1.48–1.85)	2.20 (1.96–2.46)	< 0.001	1.32 (1.28–1.37)	< 0.001
Outcome: DVT							
Number of events	552	858	1075	1408			
Persons	82,052	82,052	82,052	82,052			
Person-years	1,094,323	1,086,685	1,081,744	1,070,103			
Incidence	50.7 (46.7–55.1)	69.8 (65.0–75.0)	81.7 (76.5–87.3)	108.8 (102.7–115.3)			
Unadjusted model	Reference	1.38 (1.23–1.54)	1.62 (1.45–1.80)	2.15 (1.95–2.38)	< 0.001	1.30 (1.30–1.34)	< 0.001
Model 1	Reference	1.19 (1.07–1.33)	1.33 (1.19–1.48)	1.83 (1.65–2.03)	< 0.001	1.27 (1.23–1.31)	< 0.001
Model 2	Reference	1.19 (1.06–1.33)	1.32 (1.18–1.47)	1.80 (1.62–1.99)	< 0.001	1.26 (1.22–1.30)	< 0.001
Model 3	Reference	1.25 (1.11–1.39)	1.41 (1.26–1.59)	1.88 (1.66–2.12)	< 0.001	1.27 (1.22–1.32)	< 0.001
Sensitivity analysis 1	Reference	1.23 (1.09–1.38)	1.39 (1.24–1.57)	1.83 (1.61–2.07)	< 0.001	1.26 (1.21–1.31)	< 0.001
Sensitivity analysis 2	Reference	1.29 (1.14–1.46)	1.46 (1.29–1.66)	1.89 (1.66–2.16)	< 0.001	1.26 (1.21–1.32	< 0.001
Sensitivity analysis 3	Reference	1.32 (1.16–1.51)	1.50 (1.31–1.72)	1.99 (1.73–2.30)	< 0.001	1.28 (1.22–1.34)	< 0.001

The incidence rates are reported per 100,000 person-years with 95% CIs

Model 1: adjusted for age, sex

Model 2: adjusted for age, sex, smoking status, drinking status

Model 3: adjusted for age, sex, smoking status, drinking status, ethnicity, Townsend deprivation index, diabetes, ApoA, SBP, HbA1c, healthy diet, hypertension, LDL-C, CRP, lipid-lowering drugs, insulin, healthy physical activity, and RC

Sensitivity analysis 1: excluded follow-up time less than 1 years, remaining 327,889 participants with 8034 VTE cases, 4833 with PE cases, and 3157 with DVT cases

Sensitivity analysis 2: excluded follow-up time less than 3 years, remaining 327,135 participants with 7208 VTE cases, 4444 with PE cases, and 2826 with DVT cases

Sensitivity analysis 3: excluded follow-up time less than 5 years, remaining 326,209 participants with 6354 VTE cases, 3893 with PE cases, and 2461 with DVT cases

HRs with the corresponding 95% CIs were reported

Per SD: risk per unit increment in TyG-BMI index

Table S1 displays sensitivity, specificity, PPV, and NPV for VTE, PE, and DVT across TyG-BMI quartiles (Quartile 2, Quartile 3, Quartile 4 compared to Quartile 1). For VTE, sensitivity improved from 0.600 (95% CI 0.583–0.617) in Quartile 2 vs. Quartile 1 up to 0.705 (95% CI 0.691–0.719) in Quartile 4 vs. Quartile 1, while specificity remained around 0.502–0.505. The PPV was low across all comparisons (e.g., 0.023 in Quartile 2, 0.036 in Quartile 4), reflecting the low absolute incidence of VTE, whereas NPV was high (0.985). For PE, sensitivity ranged from 0.617 to 0.726, specificity 0.501–0.504, PPV 0.014 to 0.022, and NPV around 0.992. For DVT, sensitivity ranged from 0.577 to 0.675, specificity 0.501–0.503, PPV 0.009 to 0.014, and NPV nearly 0.993.

### Subgroup analyses

**Fig. 4:**
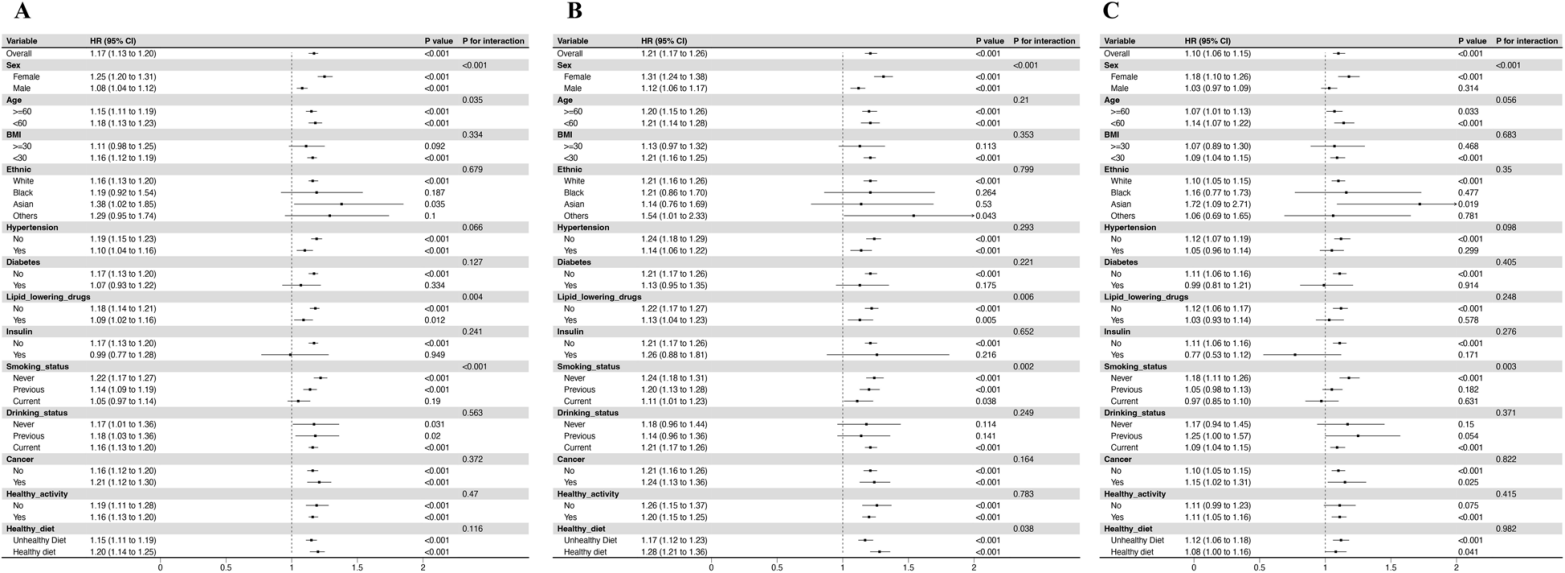
Subgroup analyses of the association between TyG-BMI index and incident outcomes. **A** TyG-BMI index in VTE, **B** TyG-BMI index in PE, **C** TyG-BMI index in DVT. The adjusted model 3 (age, sex, smoking status, drinking status, ethnicity, Townsend deprivation index, diabetes, ApoA, SBP, HbA1c, healthy diet, hypertension, LDL-C, CRP, lipid-lowering drugs, insulin use, healthy physical activity, and RC) was used in this analysis. *TyG-BMI* triglyceride-glucose body mass index

Subgroup analyses revealed stronger associations between TyG-BMI and VTE in women (HR 1.25, 95% CI 1.20–1.35) than men (HR 1.08, 95% CI 1.08–1.14; interaction *P* < 0.001). The association was also slightly stronger in participants under 60 years (HR 1.18, 95% CI 1.13–1.23) compared to those 60 and older (HR 1.15, 95% CI 1.11–1.19; *P* = 0.035). Lipid-lowering medication modified the association (interaction *P* = 0.004), with higher risk among non-users (HR 1.18, 95% CI 1.14–1.21) than users (HR 1.09, 95% CI 1.02–1.16). Smoking status demonstrated a graded interaction (*P* < 0.001); HRs were 1.22 (1.17–1.27) in never-smokers, 1.14 (1.09–1.19) in former smokers, and 1.05 (0.97–1.14) in current smokers. No significant effect modification was detected for BMI, ethnicity, hypertension, diabetes, cancer, insulin therapy, healthy activity, healthy diet, or alcohol consumption (all interaction *P* > 0.05). (Fig. 4A).

Stratified analyses for PE and DVT broadly replicated the primary findings (Fig. 4B–C). For PE, higher TyG-BMI remained deleterious across most strata, with a stronger effect seen in those with a healthy diet. No age interaction was observed (Fig. 4B). For DVT, the association was stronger in women and never-smokers, with no significant differences across the remaining subgroups (Fig. 4C).

### Population attributable fractions (PAFs) of TyG-BMI for VTE, PE, and DVT

Table S2 displays PAF estimates and 95% confidence intervals for TyG-BMI (above Quartile 1) in the overall cohort and in stratified subgroups. In the full cohort, the PAF for VTE was 0.333 (95% CI 0.295–0.371), for PE 0.376 (95% CI: 0.328–0.419), and for DVT 0.276 (95% CI: 0.212–0.341).

Among men, the PAF was 0.261 (0.198–0.326) for VTE, 0.308 (0.225–0.387)for PE, and 0.204 (0.090–0.302) for DVT; among women, PAFs were 0.365 (0.316–0.409), 0.409 (0.352–0.468), and 0.300 (0.219–0.379), respectively.

By age group, participants ≥ 60 years had PAFs of 0.333 (0.281–0.384) for VTE, 0.402 (0.337–0.462) for PE, and 0.226 (0.136–0.322) for DVT; those < 60 years had PAFs of 0.338 (0.279–0.395), 0.341 (0.263–0.412), and 0.339 (0.253–0.423). In ethnic subgroups, PAFs varied widely (e.g., for Black participants PAF 0.579 [0.327–0.819] for VTE; for White participants 0.327 [0.289–0.364]). Additional stratified PAFs by hypertension, diabetes, LLT use, smoking, and cancer status are also shown in Table S2. These subgroup patterns suggest that the proportional burden of VTE attributable to TyG-BMI differs across demographic and clinical strata.

## Discussion

In this large prospective UK Biobank cohort, a higher baseline TyG-BMI index was independently linked to a 2.10-fold greater risk of venous thromboembolism, encompassing 2.38-fold and 1.88-fold increases in PE and DVT, respectively, over a median 13.6-year follow-up. To our knowledge, this is the first study to evaluate TyG-BMI in relation to VTE risk in a general population, extending prior evidence linking metabolic syndrome to VTE [11, 13]. Our analysis suggests that TyG-BMI, a composite of BMI and the TyG index, reflects the impact of adiposity-related insulin resistance on VTE risk. Supporting this interpretation, elevated HOMA-IR, a direct measure of insulin resistance, has been independently linked to a higher incidence of VTE [13]. The TyG index alone has also been associated with thrombotic events, showing a 60% higher risk in atrial fibrillation patients and predicting in-hospital acute lower-limb DVT in intracerebral hemorrhage patients [26, 27]. The consistent observations across diverse populations strengthens the credibility of our findings and underscores the role of metabolic dysregulation, characterized by IR and obesity, in promoting thrombotic risk in both venous and arterial circulations. Prospective cohorts and meta-analyses show that individuals with obesity experience a 1.5- to twofold higher VTE risk than those of normal weight, while smaller clinical series have linked surrogate measures of IR, such as HOMA-IR, EDITH, to thrombotic events [13, 28, 29]. The TyG index—an accessible marker of IR—has been associated with cardiovascular morbidity [30, 31]. By integrating the TyG index with BMI, the TyG-BMI metric captures the combined metabolic and adiposity burden more comprehensively than either component alone [15]; nevertheless, its relation to VTE had not been examined previously. Our findings therefore extend current knowledge, demonstrating that the metabolic-obesity synergy embodied by a high TyG-BMI can predict thrombotic events many years before they become clinically manifest.

Multiple, interconnected metabolic-inflammatory pathways plausibly link an elevated TyG-BMI index with heightened VTE risk. Concomitant hyper-triglyceridemia, hyper-glycemia, and visceral adiposity drive chronic low-grade inflammation—reflected by increased interleukin-6 and tumor-necrosis-factor-α—which stimulates hepatic synthesis of fibrinogen and other coagulation factors and thereby lowers the threshold for clot formation [32–34]. In IR states, compensatory hyperinsulinemia up-regulates plasminogen-activator inhibitor-1, impairs fibrinolysis, and consolidates a hypercoagulable milieu [35, 36]. Endothelial dysfunction further skews hemostasis toward thrombosis: oxidative stress and attenuated PI3K/Akt-mediated nitric oxide signaling enhance platelet reactivity and raise circulating factor VIII activity [10, 27, 37]. The pulmonary vascular bed, densely lined with metabolically active endothelium, may be especially susceptible to these insults, offering a mechanistic basis for the stronger association we observed with pulmonary embolism than with deep-vein thrombosis [38]. Collectively, these processes explain how TyG-BMI functions as an integrated biomarker identifying individuals in whom excess adiposity and metabolic dysregulation converge to promote thrombogenesis and consequent VTE.

Restricted cubic-spline regression revealed a significant non-linear relationship between TyG-BMI and the risk of VTE (overall *P* < 0.0001; non-linearity* P* < 0.0001). The spline curve for VTE rises continuously until a TyG-BMI of 231.9, beyond which the hazard ratio still increases gradually. Notably, the increase is not abrupt. The steepness of the curve intensifies in the upper range, giving it a concave-upward shape, while the confidence bands widen substantially at extreme TyG-BMI values due to fewer observations.

A similar non-linear curvature is detected for PE (non-linearity *P* = 0.0005), and it is somewhat steeper than that for VTE. DVT exhibits a more modest non-linear pattern (non-linearity *P* = 0.0059). Biologically, this trend aligns with a “buffering” hypothesis: mild to moderate metabolic dysregulation may be offset by homeostatic mechanisms (e.g., insulin sensitivity reserve, vascular repair). But beyond some cumulative stress threshold, the effects of insulin resistance, endothelial dysfunction, low-grade inflammation, and impaired fibrinolysis may begin to accelerate more rapidly [39]. However, given the wide confidence intervals at the high tail and relatively few data points in that range, we refrain from interpreting 231.9 as a rigid threshold. Instead, we treat it as the point at which risk escalation becomes more apparent within our sample. Future work should externally validate whether a similar inflection point exists in other populations. It should also explore whether interventions that lower TyG-BMI could shift individuals back toward a safer, compensated range before overt thrombosis develops.

Notably, our results reveal several nuanced differences compared with existing studies, offering new insights. One prior cohort investigation reported that metabolic syndrome was significantly associated with VTE only in men and not in women [40], whereas another study—consistent with our findings—observed a higher proportion of women among obese VTE patients [8]. In our study, we found that elevated TyG-BMI was associated with increased VTE risk in both men and women, and although the formal sex × TyG-BMI interaction test was not significant, there was a tendency toward a somewhat greater relative risk in women compared with men. This discrepancy may stem from variations in study design and statistical power, or it may reflect the greater sensitivity of TyG-BMI as a continuous composite marker compared to the binary classification of metabolic syndrome. Our data reinforce the importance of adiposity, yet by incorporating both triglyceride and glucose concentrations, TyG-BMI additionally captures underlying insulin resistance and hyperglycemia—pathophysiological factors that anthropometric measurements alone cannot directly detect.

Interestingly, we also observed effect modification by smoking status. The association between TyG-BMI and VTE was strongest among never-smokers and attenuated in former and current smokers, which was different from a prior meta‐analysis, suggesting active smoking confer only a modest excess risk for VTE after adjustment for BMI [41]. This somewhat paradoxical trend may stem from residual confounding by sex, as women—who exhibited higher VTE risk overall—were more prevalent in the never-smoker group. These findings align with a recent Mendelian randomization analysis that identified no causal link between either current or past smoking and VTE, DVT, or PE [42]. Future research should elucidate the dose–response relationship between smoking duration and intensity and the risk of VTE.

In addition, we observed that the association between TyG-BMI and VTE appeared somewhat attenuated among participants using lipid-lowering therapy (LLT) compared to non-users. While we do not report data on the independent effects of LLT, this pattern suggests that LLT might modify—or buffer—the prothrombotic impact of adverse metabolic profiles. Supporting this notion, a recent network meta-analysis of over 45 randomized trials evaluating statins, ezetimibe, and PCSK9 inhibitors found a gradual, dose-related reduction in VTE with more intensive or combined lipid-lowering regimens [43]. While our subgroup findings are exploratory, they hint that in individuals with elevated TyG-BMI, LLT may partially mitigate excess thrombotic risk. Future studies are warranted to test whether lowering TyG-BMI or combining metabolic and lipid‐targeted therapies can reduce VTE incidence in high-risk populations.

Biologically, this observation is plausible: statins and other LLTs have known pleiotropic properties beyond lipid lowering, such as reducing systemic inflammation, downregulating PAI-1, improving endothelial function, and modulating coagulation and fibrinolysis pathways. For example, prior studies have shown that statins can reduce IL-6, CRP, and PAI-1 levels and enhance nitric oxide bioavailability, which may counteract metabolic dysfunction–driven thrombogenesis [44]. However, given that we did not present the main effects of LLT in our models, these subgroup results should be interpreted with caution. They are hypothesis-generating, indicating that in populations with high TyG-BMI, the risk conferred by metabolic dysfunction may be modifiable through therapies targeting lipid and inflammatory pathways. Future prospective or interventional studies should examine whether LLT can attenuate the incremental risk of high TyG-BMI for VTE and whether these effects differ by baseline metabolic state.

In prior population studies, the attributable fraction of VTE due to overweight and obesity was estimated at ~ 24.6% in a Norwegian cohort, providing a useful benchmark for our TyG-BMI PAFs (~ 33%) and underscoring the incremental value of integrating metabolism beyond simple adiposity [45]. Mechanistically, recent reviews posit a “metabolism-thrombosis axis,” in which dysregulated glucose and lipid metabolism, insulin resistance, oxidative stress, and endothelial dysfunction interact to promote hypercoagulability and impair fibrinolysis [46]. Thus, our PAF estimates for TyG-BMI capture the metabolic burden on VTE risk, potentially exceeding that from adiposity alone, while aligning with established dose–response data linking BMI and VTE. Similar to subgroup analyses, the attributable burden of TyG-BMI is higher among women (e.g., VTE PAF 0.365) than among men (0.261), and somewhat higher PE PAF is seen in older adults compared to younger (Table S2). Notably, in the LLT stratum, PAFs remain substantial (e.g., VTE PAF ~ 0.346 in LLT users vs. ~ 0.325 in non-users), suggesting that even among treated individuals, elevated TyG-BMI still contributes meaningfully to thrombotic risk.

### Strengths and limitations

This study represents the initial exploration of the association between TyG-BMI index and VTE, providing evidence-based support for the potential role of insulin resistance in VTE. A large sample size, a prospective research design, and a long follow-up time are the advantages of our study. Our findings were stable after excluding VTE events within the first 1, 3, and 5 years. If the TyG-BMI–VTE association were driven primarily by reverse causation or early transient triggers (e.g., surgery, hospitalization, new cancer), we would expect substantial attenuation of effect sizes with longer exclusion windows. Instead, hazard ratios changed little, the quartile dose–response persisted, and only a modest attenuation for PE at 5 years was observed—consistent with the removal of peri-operative or cancer-related PEs that peak early after diagnosis or major surgery. These observations, together with prior population-based evidence linking adiposity more strongly to PE than DVT, support a long-term metabolic–thrombotic pathway underlying the TyG-BMI association with VTE.

However, several limitations should be noted. Firstly, the observational design precludes causal inference; future Mendelian randomization studies leveraging TyG-BMI genetic instruments could address this limitation. Secondly, TyG-BMI was measured only at baseline, so regression-dilution may have attenuated the true association. Thirdly, our VTE outcome was identified using administrative ICD-10, which may lead to misclassification (false positives or false negatives). Previous validation studies report only moderate positive predictive value (≈ 72%) when using diagnosis codes alone, and improved but still imperfect accuracy even when combined with treatment or imaging codes [47, 48]. Therefore, incidence and risk estimates in our study might be biased; caution is warranted when interpreting the results, and future work with adjudicated VTE diagnoses is desirable. Finally, the cohort is predominantly White British, and replication in more ethnically diverse populations is required to establish generalizability. Future research should (i) track longitudinal changes in TyG-BMI to determine whether dynamic shifts parallel VTE risk; (ii) elucidate the biological mechanisms by which insulin resistance and obesity promote VTE; and (iii) evaluate whether targeted interventions—lifestyle modification or pharmacotherapy—can attenuate VTE risk in individuals with elevated TyG-BMI.

## Conclusion

Elevated TyG-BMI confers a substantially higher risk of first-ever VTE in the general population. These findings highlight the intertwined roles of metabolic dysfunction and obesity in venous thrombogenesis and support incorporating TyG-BMI into prevention strategies for VTE.

## Supplementary Information

Supplementary material 1. Fig. S1 Kaplan–Meier curves showing the cumulative incidence of VTE after exclusion of participants who experienced VTE within the first year of follow-up. **A** VTE, **B** PE, **C** DVT. TyG-BMI index quartile 1 was used as the reference group. *TyG-BMI* triglyceride-glucose body mass index, *DVT* deep vein thrombosis, *PE* pulmonary embolism, *VTE* venous thromboembolism.

Supplementary material 2. Fig. S2 Kaplan–Meier curves showing the cumulative incidence of VTE after exclusion of participants who experienced VTE within the first three years of follow-up. **A** VTE, **B** PE, **C** DVT. TyG-BMI index quartile 1 was used as the reference group. *TyG-BMI* triglyceride-glucose body mass index, *DVT* deep vein thrombosis, *PE* pulmonary embolism, *VTE* venous thromboembolism.

Supplementary material 3. Fig. S3 Kaplan–Meier curves showing the cumulative incidence of VTE after exclusion of participants who experienced VTE within the first five years of follow-up. **A** VTE, **B** PE, **C** DVT. TyG-BMI index quartile 1 was used as the reference group. *TyG-BMI* triglyceride-glucose body mass index, *DVT* deep vein thrombosis, *PE* pulmonary embolism, *VTE* venous thromboembolism.

Supplementary material 4.

## Data Availability

This research has been conducted using the UK Biobank Resource under application number 663576. All bona fide researchers in academic, commercial, and charitable settings could be access to the data upon application once meets the approval criteria for compensation (https://www.ukbiobank.ac.uk/register-apply).

## Abbreviations

ANOVA: One-way analysis of variance
ApoA: Apolipoprotein A1
ApoB: Apolipoprotein B
BMI: Body mass index
CI: Confidence interval
CRP: C-reactive protein
DBP: Diastolic blood pressure
DVT: Deep venous thrombosis
FBG: Fasting blood glucose
HbA1c: Hemoglobin A1c
HDL-c: High-density lipoprotein cholesterol
HR: Hazard ratio
ICD-10: International Classification of Diseases, 10th Revision
LDL-c: Low-density lipoprotein cholesterol
NPV: Negative predictive value
PAF: Population attributable fractions
PE: Pulmonary embolism
PPV: Positive predictive value
RC: Remnant cholesterol
RCS: Restricted cubic spline
SBP: Systolic blood pressure
TC: Total cholesterol
TDI: Townsend Deprivation Index
TG: Triglyceride
TyG: Triglyceride-glucose index
TyG-BMI: Triglyceride-glucose index multiplied by BMI
VTE: Venous thromboembolism

## References

[1] Heit JA. Epidemiology of venous thromboembolism. Nat Rev Cardiol. 2015;12(8):464–74. 26076949 10.1038/nrcardio.2015.83 PMC4624298

[2] Lutsey PL, Zakai NA. Epidemiology and prevention of venous thromboembolism. Nat Rev Cardiol. 2023;20(4):248–62. 36258120 10.1038/s41569-022-00787-6 PMC9579604

[3] Martin SS, Aday AW, Allen NB, Almarzooq ZI, Anderson CAM, Arora P, et al. 2025 heart disease and stroke statistics: a report of US and global data from the American Heart Association. Circulation. 2025;151(8):e41–660. 39866113 10.1161/CIR.0000000000001303 PMC12256702

[4] Zhen K, Tao Y, Xia L, Wang S, Gao Q, Wang D, et al. Epidemiology of pulmonary embolism in China, 2021: a nationwide hospital-based study. Lancet Reg Health West Pac. 2025;54:101258. 39759425 10.1016/j.lanwpc.2024.101258 PMC11699474

[5] Wendelboe A, Weitz JI. Global health burden of venous thromboembolism. Arterioscler Thromb Vasc Biol. 2024;44(5):1007–11. 38657032 10.1161/ATVBAHA.124.320151

[6] Goldhaber SZ. Venous thromboembolism: epidemiology and magnitude of the problem. Best Pract Res Clin Haematol. 2012;25(3):235–42. 22959540 10.1016/j.beha.2012.06.007

[7] Anderson FA Jr., Spencer FA. Risk factors for venous thromboembolism. Circulation. 2003;107(23 Suppl 1):I9-16. 12814980 10.1161/01.CIR.0000078469.07362.E6

[8] Weze KO, Obisesan OH, Dardari ZA, Cainzos-Achirica M, Dzaye O, Graham G, et al. The interplay of race/ethnicity and obesity on the incidence of venous thromboembolism. Am J Prev Med. 2022;63(1):e11–20. 35260291 10.1016/j.amepre.2021.12.023 PMC9232870

[9] Gregson J, Kaptoge S, Bolton T, Pennells L, Willeit P, Burgess S, et al. Cardiovascular risk factors associated with venous thromboembolism. JAMA Cardiol. 2019;4(2):163–73. 30649175 10.1001/jamacardio.2018.4537 PMC6386140

[10] Ay C, Tengler T, Vormittag R, Simanek R, Dorda W, Vukovich T, et al. Venous thromboembolism–a manifestation of the metabolic syndrome. Haematologica. 2007;92(3):374–80. 17339187 10.3324/haematol.10828

[11] Borch KH, Braekkan SK, Mathiesen EB, Njolstad I, Wilsgaard T, Stormer J, et al. Abdominal obesity is essential for the risk of venous thromboembolism in the metabolic syndrome: the Tromso study. J Thromb Haemost. 2009;7(5):739–45. 19036065 10.1111/j.1538-7836.2008.03234.x

[12] Bluher M. Metabolically healthy obesity. Endocr Rev. 2020. 10.1210/endrev/bnaa004. 32128581 10.1210/endrev/bnaa004 PMC7098708

[13] Van Schouwenburg IM, Mahmoodi BK, Veeger NJ, Bakker SJ, Kluin-Nelemans HC, Meijer K, et al. Insulin resistance and risk of venous thromboembolism: results of a population-based cohort study. J Thromb Haemost. 2012;10(6):1012–8. 22443091 10.1111/j.1538-7836.2012.04707.x

[14] Yang Z, Gong H, Kan F, Ji N. Association between the triglyceride glucose (TyG) index and the risk of acute kidney injury in critically ill patients with heart failure: analysis of the MIMIC-IV database. Cardiovasc Diabetol. 2023;22(1):232. 37653418 10.1186/s12933-023-01971-9 PMC10472684

[15] Li W, Shen C, Kong W, Zhou X, Fan H, Zhang Y, et al. Association between the triglyceride glucose-body mass index and future cardiovascular disease risk in a population with Cardiovascular-Kidney-Metabolic syndrome stage 0-3: a nationwide prospective cohort study. Cardiovasc Diabetol. 2024;23(1):292. 39113004 10.1186/s12933-024-02352-6 PMC11308445

[16] Er LK, Wu S, Chou HH, Hsu LA, Teng MS, Sun YC, et al. Triglyceride glucose-body mass index is a simple and clinically useful surrogate marker for insulin resistance in nondiabetic individuals. PLoS ONE. 2016;11(3):e0149731. 26930652 10.1371/journal.pone.0149731 PMC4773118

[17] Wang M, Chang M, Shen P, Wei W, Li H, Shen G. Application value of triglyceride-glucose index and triglyceride-glucose body mass index in evaluating the degree of hepatic steatosis in non-alcoholic fatty liver disease. Lipids Health Dis. 2023;22(1):186. 37924128 10.1186/s12944-023-01954-5 PMC10623715

[18] Nikbakht HR, Najafi F, Shakiba E, Darbandi M, Navabi J, Pasdar Y. Triglyceride glucose-body mass index and hypertension risk in Iranian adults: a population-based study. BMC Endocr Disord. 2023;23(1):156. 37479987 10.1186/s12902-023-01411-5 PMC10360216

[19] Zhang Z, Zhao L, Lu Y, Meng X, Zhou X. Association between non-insulin-based insulin resistance indices and cardiovascular events in patients undergoing percutaneous coronary intervention: a retrospective study. Cardiovasc Diabetol. 2023;22(1):161. 37386494 10.1186/s12933-023-01898-1 PMC10311786

[20] Ay C, Posch F, Kaider A, Zielinski C, Pabinger I. Estimating risk of venous thromboembolism in patients with cancer in the presence of competing mortality. J Thromb Haemost. 2015;13(3):390–7. 25529107 10.1111/jth.12825 PMC7279950

[21] Rassy N, Van Straaten A, Carette C, Hamer M, Rives-Lange C, Czernichow S. Association of healthy lifestyle factors and obesity-related diseases in adults in the UK. JAMA Netw Open. 2023;6(5):e2314741. 37234008 10.1001/jamanetworkopen.2023.14741 PMC10220514

[22] Lloyd-Jones DM, Hong Y, Labarthe D, Mozaffarian D, Appel LJ, Van Horn L, et al. Defining and setting national goals for cardiovascular health promotion and disease reduction: the American Heart Association’s strategic Impact Goal through 2020 and beyond. Circulation. 2010;121(4):586–613. 20089546 10.1161/CIRCULATIONAHA.109.192703

[23] Austin PC. Graphical methods to illustrate the nature of the relation between a continuous variable and the outcome when using restricted cubic splines with a Cox proportional hazards model. Stat Methods Med Res. 2025;34(2):277–85. 39431319 10.1177/09622802241287707 PMC11874503

[24] Strain T, Wijndaele K, Sharp SJ, Dempsey PC, Wareham N, Brage S. Impact of follow-up time and analytical approaches to account for reverse causality on the association between physical activity and health outcomes in UK Biobank. Int J Epidemiol. 2020;49(1):162–72. 31651957 10.1093/ije/dyz212 PMC7124507

[25] Greenland S, Drescher K. Maximum likelihood estimation of the attributable fraction from logistic models. Biometrics. 1993;49(3):865–72. 8241375

[26] Rivera-Caravaca JM, Soler-Espejo E, Gonzalez-Lozano E, Lip GYH, Marin F, Roldan V. The triglyceride-glucose index is a marker of thromboembolic events in atrial fibrillation patient under oral anticoagulation therapy. Eur Heart J. 2024. 10.1093/eurheartj/ehae666.587.

[27] Zhang H, Huang L, Li F. A nomogram based on the TyG index for the prediction of lower-limb venous thrombosis in patients with intracerebral hemorrhage. Sci Rep. 2025;15(1):17406. 40389507 10.1038/s41598-025-01923-1 PMC12089349

[28] Delluc A, De Moreuil C, Kerspern H, Le Moigne E, Mottier D, Tromeur C, et al. Body mass index, a major confounder to insulin resistance association with unprovoked venous thromboembolism. Results from the EDITH case-control study. Thromb Haemost. 2013;110(3):593–7. 23803721 10.1160/TH13-01-0048

[29] Ageno W, Di Minno MN, Ay C, Jang MJ, Hansen JB, Steffen LM, et al. Association between the metabolic syndrome, its individual components, and unprovoked venous thromboembolism: results of a patient-level meta-analysis. Arterioscler Thromb Vasc Biol. 2014;34(11):2478–85. 25212233 10.1161/ATVBAHA.114.304085 PMC4322778

[30] Dang K, Wang X, Hu J, Zhang Y, Cheng L, Qi X, et al. The association between triglyceride-glucose index and its combination with obesity indicators and cardiovascular disease: NHANES 2003-2018. Cardiovasc Diabetol. 2024;23(1):8. 38184598 10.1186/s12933-023-02115-9 PMC10771672

[31] Zhang Q, Xiao S, Jiao X, Shen Y. The triglyceride-glucose index is a predictor for cardiovascular and all-cause mortality in CVD patients with diabetes or pre-diabetes: evidence from NHANES 2001-2018. Cardiovasc Diabetol. 2023;22(1):279. 37848879 10.1186/s12933-023-02030-z PMC10583314

[32] Saltiel AR, Olefsky JM. Inflammatory mechanisms linking obesity and metabolic disease. J Clin Invest. 2017;127(1):1–4. 28045402 10.1172/JCI92035 PMC5199709

[33] Oikonomou E, Leopoulou M, Theofilis P, Antonopoulos AS, Siasos G, Latsios G, et al. A link between inflammation and thrombosis in atherosclerotic cardiovascular diseases: clinical and therapeutic implications. Atherosclerosis. 2020;309:16–26. 32858395 10.1016/j.atherosclerosis.2020.07.027

[34] Tang H, Cheng Z, Li N, Mao S, Ma R, He H, et al. The short- and long-term associations of particulate matter with inflammation and blood coagulation markers: a meta-analysis. Environ Pollut. 2020;267:115630. 33254709 10.1016/j.envpol.2020.115630 PMC7687019

[35] Bilgic Gazioglu S, Akan G, Atalar F, Erten G. PAI-1 and TNF-alpha profiles of adipose tissue in obese cardiovascular disease patients. Int J Clin Exp Pathol. 2015;8(12):15919–25. 26884864 PMC4730077

[36] Altalhi R, Pechlivani N, Ajjan RA. PAI-1 in diabetes: pathophysiology and role as a therapeutic target. Int J Mol Sci. 2021. 10.3390/ijms22063170. 33804680 10.3390/ijms22063170 PMC8003717

[37] Muniyappa R, Sowers JR. Role of insulin resistance in endothelial dysfunction. Rev Endocr Metab Disord. 2013;14(1):5–12. 23306778 10.1007/s11154-012-9229-1 PMC3594115

[38] Willson C, Watanabe M, Tsuji-Hosokawa A, Makino A. Pulmonary vascular dysfunction in metabolic syndrome. J Physiol. 2019;597(4):1121–41. 30125956 10.1113/JP275856 PMC6375868

[39] Del Turco S, Gaggini M, Daniele G, Basta G, Folli F, Sicari R, et al. Insulin resistance and endothelial dysfunction: a mutual relationship in cardiometabolic risk. Curr Pharm Des. 2013;19(13):2420–31. 23173591 10.2174/1381612811319130010

[40] Steffen LM, Cushman M, Peacock JM, Heckbert SR, Jacobs DR Jr., Rosamond WD, et al. Metabolic syndrome and risk of venous thromboembolism: longitudinal investigation of thromboembolism etiology. J Thromb Haemost. 2009;7(5):746–51. 19175496 10.1111/j.1538-7836.2009.03295.x PMC2810102

[41] Cheng YJ, Liu ZH, Yao FJ, Zeng WT, Zheng DD, Dong YG, et al. Current and former smoking and risk for venous thromboembolism: a systematic review and meta-analysis. PLoS Med. 2013;10(9):e1001515. 24068896 10.1371/journal.pmed.1001515 PMC3775725

[42] Du HC, Zheng YF, Shen MQ, Deng BY. No genetic causality between tobacco smoking and venous thromboembolism: a two-sample Mendelian randomization study. Thromb Haemost. 2024;124(8):795–802. 38387601 10.1055/s-0044-1781425

[43] Farmakis IT, Christodoulou KC, Hobohm L, Konstantinides SV, Valerio L. Lipid lowering for prevention of venous thromboembolism: a network meta-analysis. Eur Heart J. 2024;45(35):3219–27. 38874212 10.1093/eurheartj/ehae361

[44] Wang Z, Zhang P, Tian J, Zhang P, Yang K, Li L. Statins for the primary prevention of venous thromboembolism. Cochrane Database Syst Rev. 2024;11(11):CD014769. 39498835 10.1002/14651858.CD014769.pub2 PMC11536507

[45] Frischmuth T, Tondel BG, Braekkan SK, Hansen JB, Morelli VM. The risk of incident venous thromboembolism attributed to overweight and obesity: the Tromso study. Thromb Haemost. 2024;124(3):239–49. 37549694 10.1055/s-0043-1772212

[46] Cao Z, Jiang X, He Y, Zheng X. Metabolic landscape in venous thrombosis: insights into molecular biology and therapeutic implications. Ann Med. 2024;56(1):2401112. 39297312 10.1080/07853890.2024.2401112 PMC11413966

[47] Sanfilippo KM, Wang TF, Gage BF, Liu W, Carson KR. Improving accuracy of International classification of diseases codes for venous thromboembolism in administrative data. Thromb Res. 2015;135(4):616–20. 25613924 10.1016/j.thromres.2015.01.012 PMC4361236

[48] Fang MC, Fan D, Sung SH, Witt DM, Schmelzer JR, Steinhubl SR, et al. Validity of using inpatient and outpatient administrative codes to identify acute venous thromboembolism: the CVRN VTE study. Med Care. 2017;55(12):e137–43. 29135777 10.1097/MLR.0000000000000524 PMC5125903

