# Supplementary figures and images for: Triglyceride-glucose body mass index and risk of incident venous thromboembolism: a prospective cohort study from the UK Biobank
Source: Eur J Med Res. 2026 Jan 10;31:246. doi: 10.1186/s40001-025-03824-5 (PMC12882130; doi:10.1186/s40001-025-03824-5)

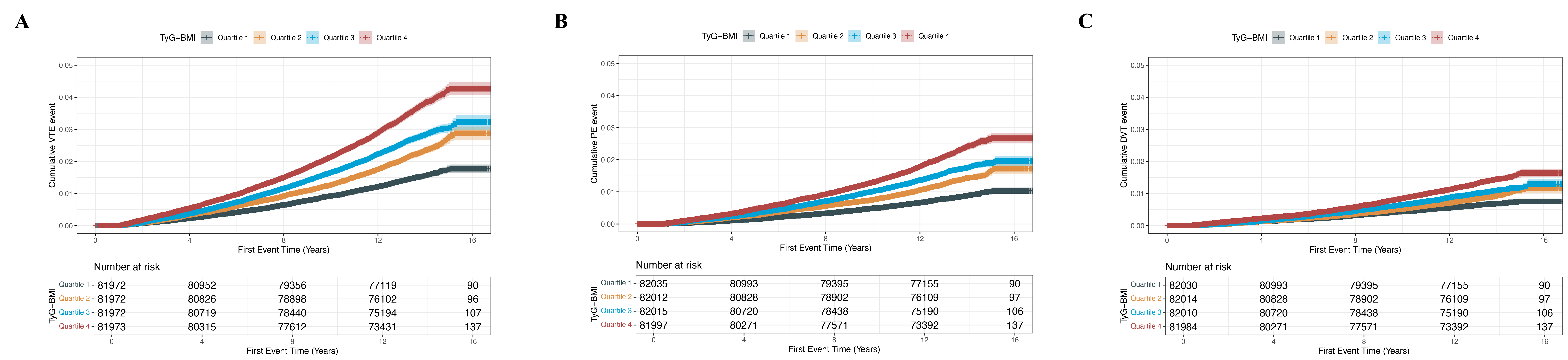

Supplement: Supplementary file 1 — Supplementary material 1. Fig. S1 Kaplan–Meier curves showing the cumulative incidence of VTE after exclusion of participants who experienced VTE within the first year of follow-up. A VTE, B PE, C DVT. TyG-BMI index quartile 1 was used as the reference group. TyG-BMI triglyceride-glucose body mass index, DVT deep vein thrombosis, PE pulmonary embolism, VTE venous thromboembolism. [file 40001_2025_3824_MOESM1_ESM.tif]

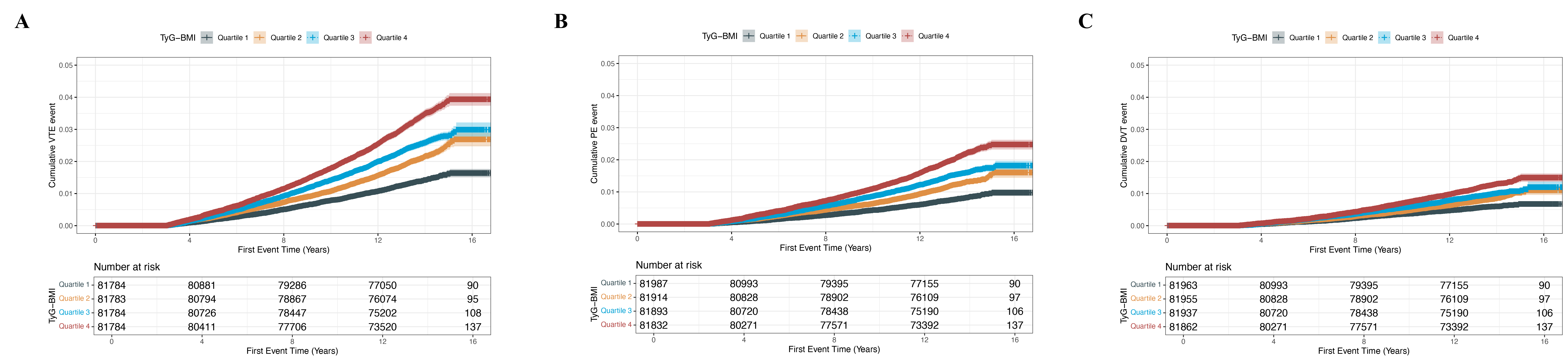

Supplement: Supplementary file 2 — Supplementary material 2. Fig. S2 Kaplan–Meier curves showing the cumulative incidence of VTE after exclusion of participants who experienced VTE within the first three years of follow-up. A VTE, B PE, C DVT. TyG-BMI index quartile 1 was used as the reference group. TyG-BMI triglyceride-glucose body mass index, DVT deep vein thrombosis, PE pulmonary embolism, VTE venous thromboembolism. [file 40001_2025_3824_MOESM2_ESM.tif]

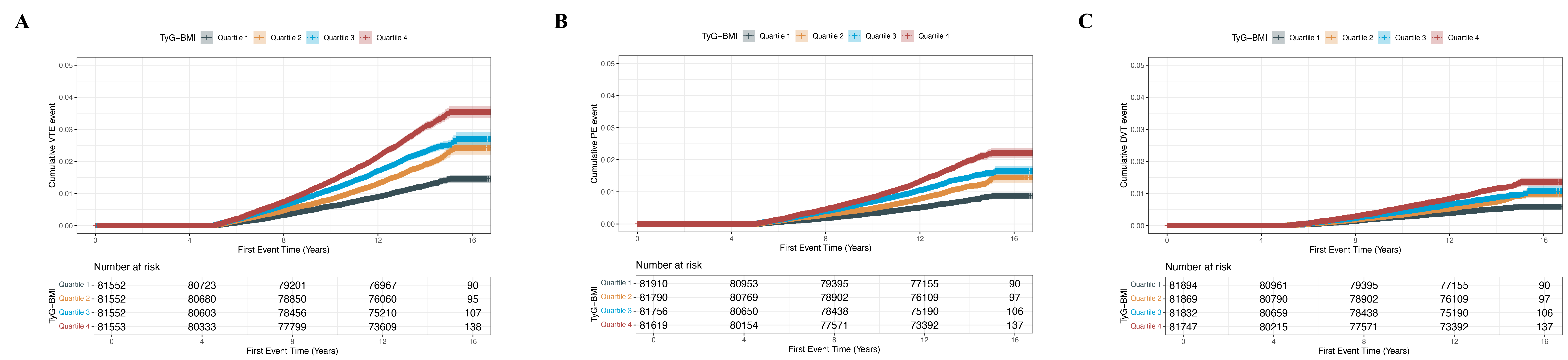

Supplement: Supplementary file 3 — Supplementary material 3. Fig. S3 Kaplan–Meier curves showing the cumulative incidence of VTE after exclusion of participants who experienced VTE within the first five years of follow-up. A VTE, B PE, C DVT. TyG-BMI index quartile 1 was used as the reference group. TyG-BMI triglyceride-glucose body mass index, DVT deep vein thrombosis, PE pulmonary embolism, VTE venous thromboembolism. [file 40001_2025_3824_MOESM3_ESM.tif]
